# In Vitro Antiviral Effect and Potential Neuroprotection of *Salvadora persica* L. Stem Bark Extract against Lipopolysaccharides-Induced Neuroinflammation in Mice: LC-ESI-MS/MS Analysis of the Methanol Extract

**DOI:** 10.3390/ph16030398

**Published:** 2023-03-06

**Authors:** Reem Binsuwaidan, Walaa A. Negm, Engy Elekhnawy, Nashwah G. M. Attallah, Eman Ahmed, Sameh Magdeldin, Ehssan Moglad, Sally Abdallah Mostafa, Suzy A. El-Sherbeni

**Affiliations:** 1 Department of Pharmaceutical Science, College of Pharmacy, Princess Nourah Bint Abdulrahman University, P.O. Box 84428, Riyadh 11671, Saudi Arabia; 2 Department of Pharmacognosy, Faculty of Pharmacy, Tanta University, Tanta 31527, Egypt; 3 Department of Pharmaceutical Microbiology, Faculty of Pharmacy, Tanta University, Tanta 31527, Egypt; 4 The Egyptian Drug Authority (EDA), Previously NODCAR, Giza 8655, Egypt; 5 Department of Pharmacology, Faculty of Veterinary Medicine, Suez Canal University, Ismailia 41522, Egypt; 6 Proteomics and Metabolomics Research Program, Department of Basic Research, Children’s Cancer Hospital 57357, Cairo 11441, Egypt; 7 Department of Physiology, Faculty of Veterinary Medicine, Suez Canal University, Ismailia 41522, Egypt; 8 Department of Pharmaceutics, College of Pharmacy, Prince Sattam Bin Abdulaziz University, P.O. Box 173, Al-Kharj 11942, Saudi Arabia; 9 Department of Medical Biochemistry and Molecular Biology, Faculty of Medicine, Mansoura University, Mansoura 35516, Egypt

**Keywords:** brain, HSV-2, metabolomics, neuroinflammation, oxidative stress, phytoconstituents

## Abstract

Neuroinflammation is a serious immunomodulatory complex disorder that causes neurological and somatic ailments. The treatment of brain inflammation with new drugs derived from natural sources is a significant therapeutic goal. Utilizing LC-ESI-MS/MS analysis, the active constituents of *Salvadora persica* extract (SPE) were identified tentatively as exerting antioxidant and anti-inflammatory effects in natural medicine. Herein, we determined the antiviral potential of SPE against herpes simplex virus type 2 (HSV-2) using the plaque assay. HSV-2 is a neurotropic virus that can cause neurological diseases. SPE exhibited promising antiviral potential with a half-maximal cytotoxic concentration (CC_50_) of 185.960 ± 0.1 µg/mL and a half-maximal inhibitory concentration (IC_50_) of 8.946 ± 0.02 µg/mL. The in vivo study of the SPE impact against lipopolysaccharide (LPS)-induced neuroinflammation was performed using 42 mice divided into seven groups. All groups were administered LPS (0.25 mg/kg) intraperitoneally, except for the normal and SPE groups 1 and 2. Groups 5, 6, and 7 received 100, 200, and 300 mg/kg SPE. It was revealed that SPE inhibited acetylcholinesterase in the brain. It increased superoxide dismutase and catalase while decreasing malondialdehyde, which explains its antioxidative stress activity. SPE downregulated the gene expression of the inducible nitric oxide synthase, as well as the apoptotic markers (caspase-3 and c-Jun). In addition, it decreased the expression of the proinflammatory cytokines (interleukin-6 and tumor necrosis factor-alpha). Mice administered SPE (300 mg/kg) with LPS exhibited normal neurons in the cerebral cortices, hippocampus pyramidal layer, and cerebellum, as determined by the histopathological analysis. Therefore, using *S. persica* to prevent and treat neurodegeneration could be a promising new therapeutic strategy to be explored.

## 1. Introduction

Neuroinflammation is a significant factor that causes many central nervous system (CNS) ailments. It is a complex response to neuronal cell injuries in the brain, including the stimulation of microglia, which are the major cellular components of neuroinflammation. This condition is characterized by the release of proinflammatory cytokines, such as interleukin-6 (IL-6) and tumor necrosis factor-alpha (TNFα), as well as the production of reactive oxygen species (ROS) and nitric oxide (NO). Neuroinflammation is an innate immune response that acts against infections and variable pathogens. Neurodegenerative and other CNS diseases, including Alzheimer’s disease (AD) and Parkinson’s (PA), are caused by the proinflammatory mediators mentioned earlier through their contribution to the death of neurons. It is a significant target for treating the neuroinflammation of the brain to manage the severe manifestations of different CNS diseases [1,2,3].

Arak shrubs belong to the Salvadoraceae family, also known as *Salvadora persica* L. [4,5]. The shrubs are disseminated across the Arabian Peninsula, Africa, and India [6]. It is known that the stems, bark, and roots contain variable secondary metabolites with remarkable free radical scavenging and anti-inflammatory impacts [7]. *S. persica* contains essential oils [8]; flavonoids (such as rutin and quercetin) [9,10]; phenolic acids (such as gallic, chlorogenic, caffeic, and rosmarinic acids) [11]; alkaloids such as persicaline [12]; sulfated glycosides [13]; phenolic diterpenes; salvadourea; benzyl isothiocyanate [14]; chlorides; fluorides; tannins; and fatty acids [9]. It played a significant role in preventing and treating different dental diseases [15]. In addition, it exhibited variable biological activities such as wound healing, analgesic, antimicrobial, antidepressant, anticonvulsant, and anticancer potentials [9]. To discover new drugs for managing neuroinflammation in the various CNS diseases, it is essential to investigate the effects of natural substances such as plants. Interestingly, several plant extracts were found to be effective against the neurodegeneration of AD and PA diseases [16,17].

Many neurotropic viruses can infect the CNS. These viruses, such as herpesviruses, Ebola, and rabies, can lead to multiple nervous system diseases, including meningitis, encephalitis, and flaccid paralysis [18]. Herpesviruses, such as herpes simplex virus type 1 (HSV-1) and HSV-2, are enveloped viruses with double-stranded DNA. They primarily infect the hosts’ skin, mucous membranes, and nerve tissues, resulting in high morbidity and mortality rates [19]. Due to their latent neuron infections, HSV-1 and HSV-2 have a high prevalence in the human population, with lifelong infections and sporadic recurrences [20]. Variants of HSV that show resistance to the currently used antivirals are increasing, particularly in immunocompromised patients [21]. Therefore, it is crucial to discover novel antivirals that can decrease the severity and duration of the infections caused by such viruses. In this study, we aimed to investigate the antiviral activity of *Salvadora persica* methanol extract of stem bark (SPE) against the HSV-2 virus.

Since there are insufficient treatments for neurodegenerative disorders, investigating the possibility of using plant extracts as safe and inexpensive sources to prevent and treat such disorders is a promising idea that must be investigated. The present study aimed to investigate the activity of SPE against lipopolysaccharide (LPS)-induced neuroinflammation in mice and assess the underlying mechanism of action and the antiviral activity of SPE against the neurotropic virus HSV-2. In addition, the LC-ESI-MS/MS technique was used to tentatively identify the phytochemical constituents of SPE.

## 2. Results

### 2.1. Recognition of the Chemical Profile of Salvadora persica Extract by LC-ESI-MS/MS Analysis

**Figure 1:**
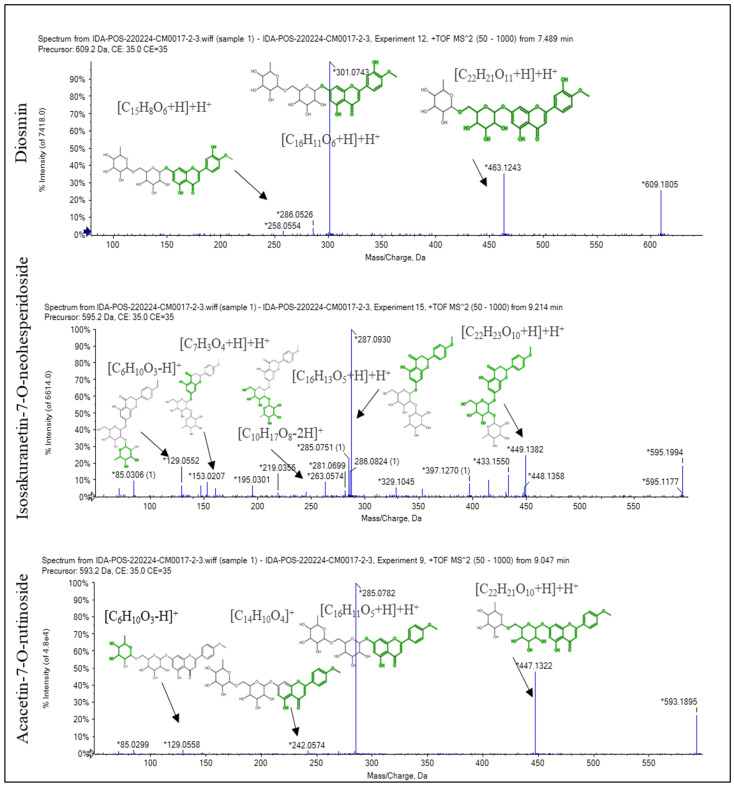
The fragmentation pattern of the main compounds recognized in the methanol extract of *Salvadora persica* stem bark: diosmin, isosakuranetin-7-*O*-neohesperidoside, and acacetin-7-*O*-rutinoside. * stands for *m*/*z* of [M + H]^+^, [M − H]^−^, and fragments of each compound.

**Figure 2:**
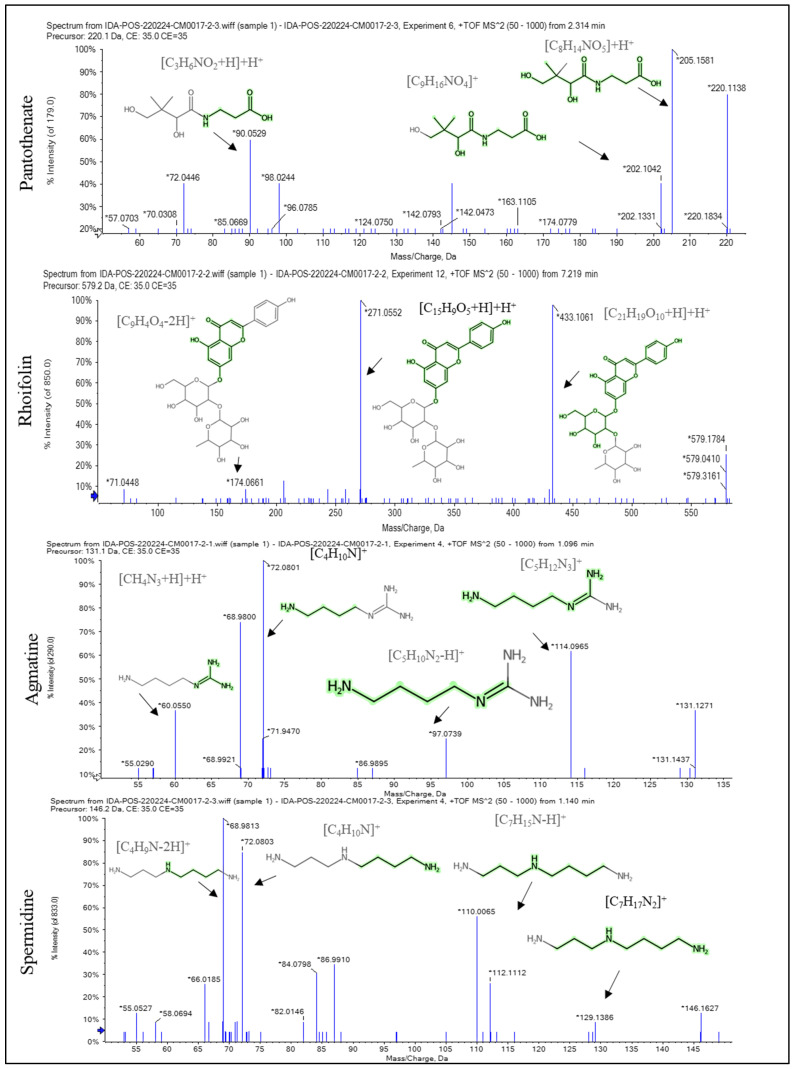
The fragmentation pattern of the main compounds recognized in the methanol extract of *Salvadora persica* stem bark: pantothenate, rhoifolin, agmatine, and spermidine. * stands for *m*/*z* of [M + H]^+^, [M − H]^−^, and fragments of each compound.

**Figure 3:**
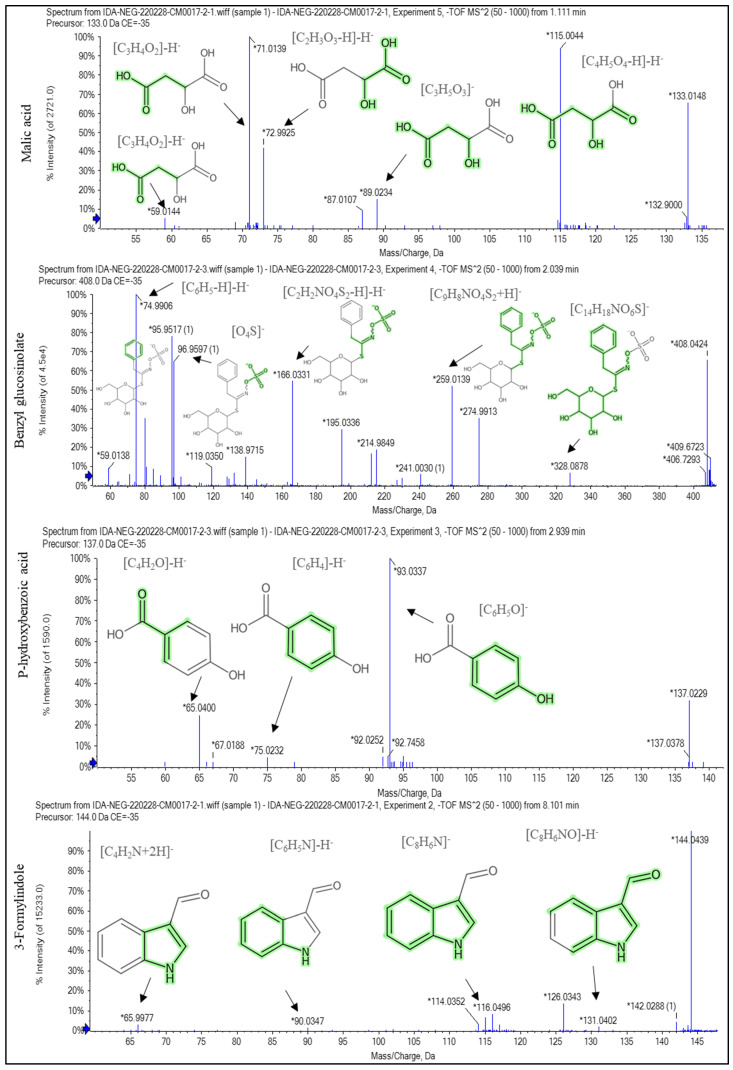
The fragmentation pattern of the main compounds recognized in the methanol extract of *Salvadora persica* stem bark: malic acid, benzyl glucosinolate, p-hydroxy benzoic acid, and 3-formylindole. * stands for *m*/*z* of [M + H]^+^, [M − H]^−^, and fragments of each compound.

The LC-ESI-MS/MS analysis of SPE could represent the phytochemical components and tentatively demonstrate the diversity of its compounds. The presence of phenolic glycosides such as syringin; coumaric acids derivatives (such as rosmarinic acid); phenolic acids (such as sinapic acid); alkyl glucosinolate (such as benzyl glucosinolate); flavonoids (such as kaempferol-7-*O*-neohesperidoside, hesperetin, rhoifolin, and apigenin); and indoles (such as 3-formylindole) displayed the diversity of the components. The major compounds displayed in negative and positive ESI modes were diosmin, isosakuranetin-7-*O*-neohesperidoside, acacetin-7-*O*-rutinoside, rhoifolin, agmatine, benzyl glucosinolate, hydroxybenzoic acid, spermidine, pantothenate, malic acid, and 3-formylindole, as displayed in Table 1 and Figure 1, Figure 2 and Figure 3. Figures S1 and S2 depict the negative and positive modes of the total ion chromatogram, respectively.

### 2.2. Antiviral Activity

#### 2.2.1. Cytotoxicity of SPE on the Vero-E6 Cells

**Figure 4:**
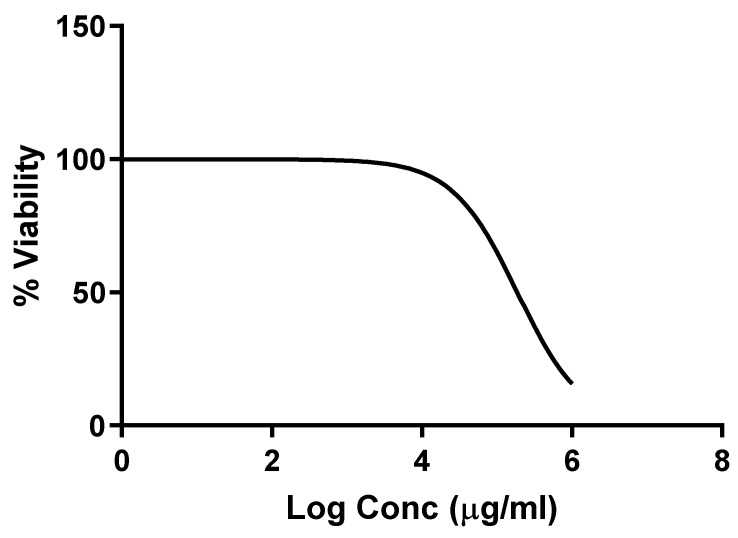
Cytotoxicity curve of *Salvadora persica* methanol extract (CC_50_ of 185.960 ± 0.1 µg/mL). The results are presented as the mean ± SD.

The values of CC_50_ of SPE on Vero-E6 cells were determined by the MTT assay, as demonstrated in Figure 4. The value of CC_50_ was 185.960 ± 0.1 µg/mL. This means that the concentration of SPE that caused death for 50% of the Vero-E6 cells was equal to 185.960 ± 0.1 µg/mL. Thus, we used lower concentrations studying the antiviral activity of SPE on Vero-E6 cells infected with the HSV-2 virus.

#### 2.2.2. Antiviral Activity of SPE

**Figure 5:**
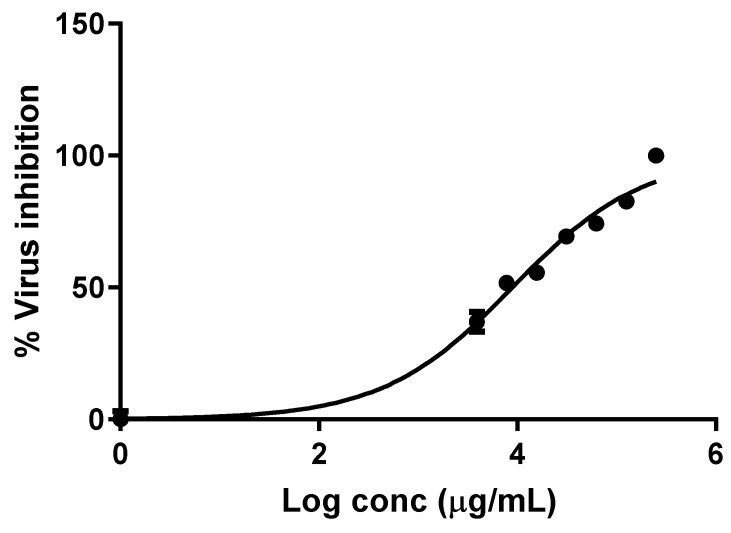
Antiviral activity of *Salvadora persica* methanol extract (IC_50_ of 8.946 ± 0.02 µg/mL) against the HSV-2 virus. The results are presented as the mean ± SD.

The antiviral potential of the SPE was explored against the HSV-2 virus, and the value of the half-maximal inhibitory concentration (IC_50_) was determined, as shown in Figure 5. The value of IC_50_ of SPE was 8.946 ± 0.02 µg/mL, and this concentration was needed to inhibit the HSV-2 virus by 50%.

### 2.3. In Vivo Study

#### 2.3.1. Effect on the AChE Activity

**Figure 6:**
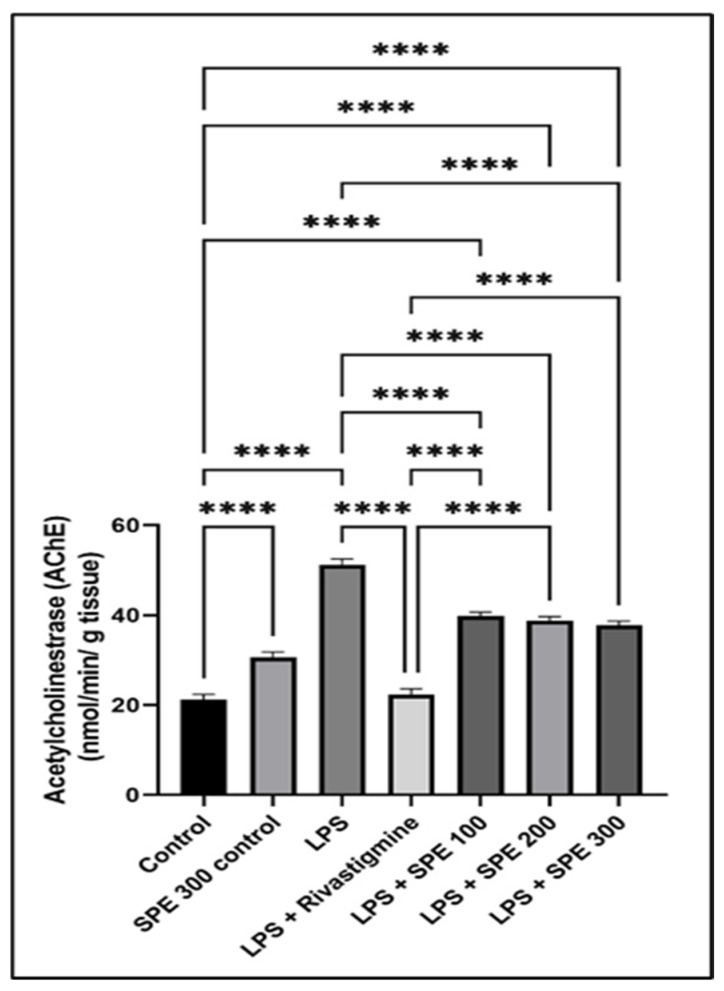
The acetylcholinesterase (AChE) activity of the control (group 1), SPE 300 control (group 2), LPS (group 3), LPS + Rivastigmine (group 4), LPS + SPE 100 (group 5), LPS + SPE 200 (group 6), and LPS + SPE 300 (group 7). ****: Significant at *p* ≤ 0.0001.

LPS significantly increased (*p* < 0.05) the AChE activity compared to the vehicle control. Figure 6 demonstrates that pretreatment with SPE significantly decreased (*p* < 0.05) the increased AChE activity in the brain compared to the LPS-treated group. Groups 5, 6, and 7 revealed a significant decrease (*p* < 0.05) in the AChE activity with percentages of 23.07%, 25%, and 26.9%, respectively.

#### 2.3.2. Effect of SPE on the LPS-Induced Oxidative Stress Markers

**Figure 7:**
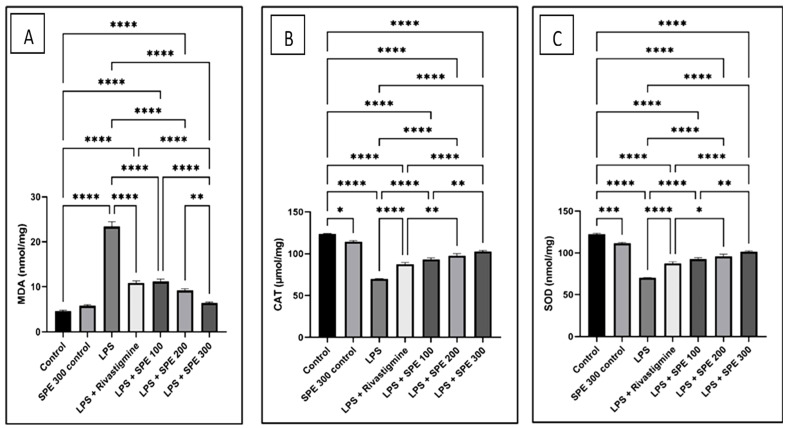
The levels of (**A**) malondialdehyde (MDA), (**B**) catalase (CAT), and (**C**) superoxide dismutase (SOD) of the control (group 1), SPE 300 control (group 2), LPS (group 3), LPS + Rivastigmine (group 4), LPS + SPE 100 (group 5), LPS + SPE 200 (group 6), and LPS + SPE 300 (group 7). *: Significant at *p* ≤ 0.05. **: Significant at *p* ≤ 0.01. ***: Significant at *p* ≤ 0.001. ****: Significant at *p* ≤ 0.0001.

Compared to the vehicle control group, LPS significantly increased (*p* < 0.05) the MDA levels, whereas Rivastigmine (RVS) and SPE significantly decreased (*p* < 0.05) the LPS-induced increase in the MDA levels (Figure 7).

The level of oxidative stress induced by LPS administration was evaluated by the CAT and SOD levels in the brain. The levels of CAT and SOD in the LPS-treated group were significantly lower (*p* < 0.05) than the vehicle control group. Compared to the LPS-treated group, the pretreatment with RVS and SPE restored the decreased levels of SOD and CAT (Figure 7).

#### 2.3.3. Effect of SPE on the Relative Gene Expression of IL-6, TNF-α, and iNOS

**Figure 8:**
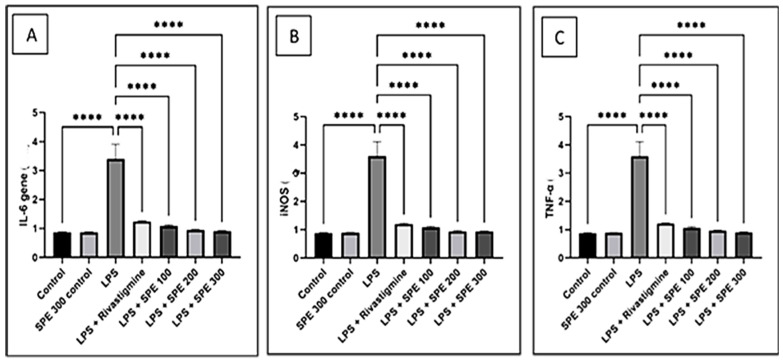
The relative gene expression of (**A**) interleukin-6 (IL-6), (**B**) inducible nitric oxide synthase (iNOS), and (**C**) tumor necrosis factor-alpha (TNF-α) of the control (group 1), SPE 300 control (group 2), LPS (group 3), LPS + Rivastigmine (group 4), LPS + SPE 100 (group 5), LPS + SPE 200 (group 6), and LPS + SPE 300 (group 7). ****: Significant at *p* ≤ 0.0001.

Compared to the vehicle-treated group, IL-6 gene expression was significantly increased in the LPS-treated group (*p* < 0.05). The significantly low expression levels (*p* < 0.05) of IL-6 in the RVS and SPE pretreated group demonstrated a substantial anti-inflammatory effect in comparison to the LPS-treated group (Figure 8).

Furthermore, the TNF-α and iNOS expression levels were significantly elevated (*p* < 0.05) in the LPS-treated group compared to the vehicle control group. The increased expression of the TNF- α and iNOS levels by LPS was significantly downregulated (*p* < 0.05) by the RVS and SPE pretreatment (Figure 8).

#### 2.3.4. Effect of SPE on the Proapoptotic Gene Expression

In comparison with the vehicle control group, the administration of LPS dramatically increased (*p* < 0.05) the caspase-3 expression level. The increase in the caspase-3 expression levels induced by LPS was considerably reduced (*p* < 0.05) by the RVS and SPE pretreatment.

**Figure 9:**
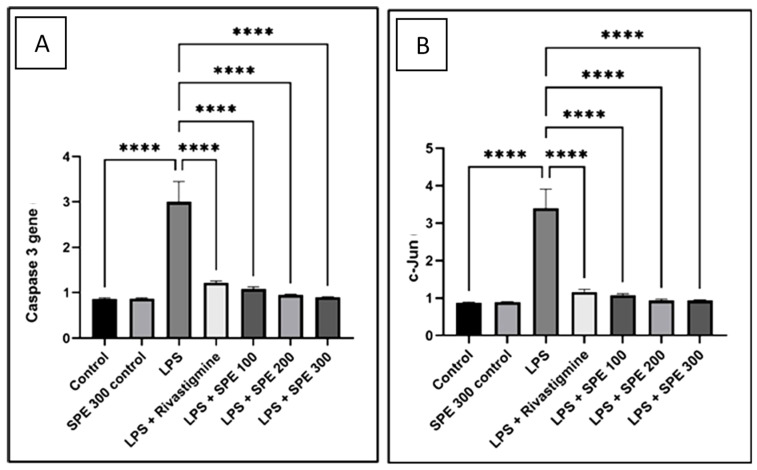
The relative gene expression of (**A**) caspase-3 and (**B**) c-Jun of the control (group 1), SPE 300 control (group 2), LPS (group 3), LPS + Rivastigmine (group 4), LPS + SPE 100 (group 5), LPS + SPE 200 (group 6), and LPS + SPE 300 (group 7). **** Significant at *p* ≤ 0.0001.

The c-Jun expression level was substantially higher (*p* < 0.05) after exposure to LPS than in the vehicle control group. Additionally, the c-Jun expression levels were significantly lower (*p* < 0.05) in the RVS and SPE groups (Figure 9).

#### 2.3.5. Histopathological Data

**Figure 10:**
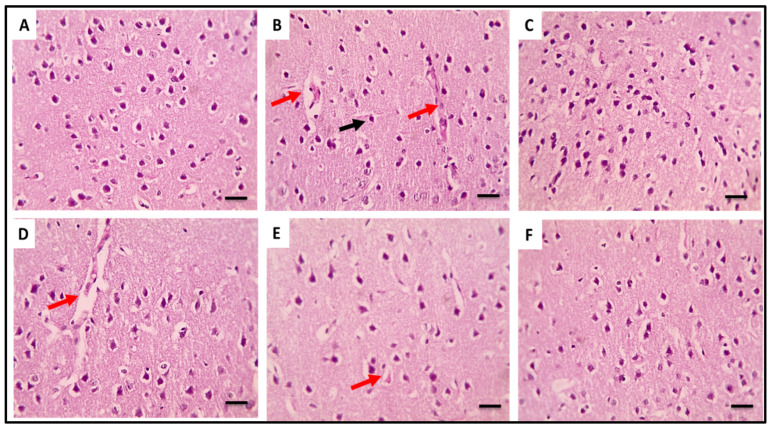
H&E-stained brain sections showing the cerebral cortices of the different groups. Normal neurons were revealed in the control group (group 1, (**A**)). Sections from the group that received LPS (group 3, (**B**)) revealed congested blood vessels (red arrows) and the shrinkage of neurons (black arrows). Sections from the treated group with RVS (group 4, (**C**)) showed normal neurons. Sections from the treated group with SPE 100 mg/kg (group 5, (**D**)) showed normal neurons with congested blood vessels (red arrow). Sections from the treated group with SPE 200 mg/kg (group 6, (**E**)) showed normal neurons with mildly congested blood vessels (red arrow). Sections from the treated group with SPE 300 mg/kg (group 7, (**F**)) showed normal neurons (×400).

**Figure 11:**
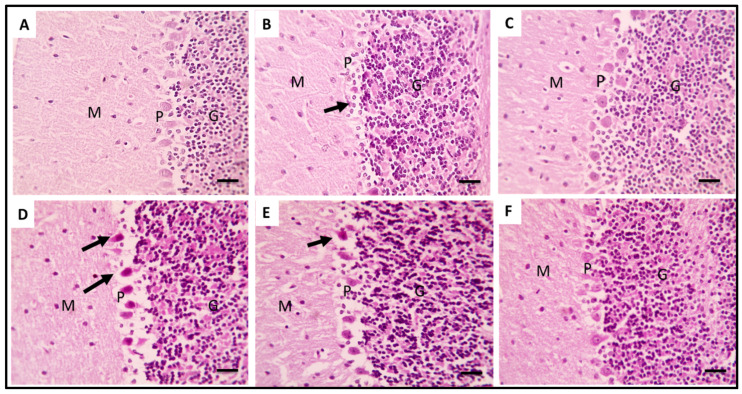
H&E-stained brain sections showing the cerebellum of the different groups. Normal neurons were revealed in the control group (group 1, (**A**)). Sections from the group that received LPS (group 3, (**B**)) showed a severe loss of purkinje neurons (black arrow). Sections from the treated group with RVS (group 4, (**C**)) showed normal purkinje neurons. Sections from the treated group with SPE 100 mg/kg (group 5, (**D**)) showed markedly degenerated purkinje neurons (black arrows). Sections from the treated group with SPE 200 mg/kg (group 6, (**E**)) showed a few degenerated purkinje neurons (black arrow). Sections from the treated group with SPE 300 mg/kg (group 7, (**F**)) showed normal purkinje neurons (×400). The letters G, P, and M stand for granular layer, purkinje layer, and molecular layer, respectively.

**Figure 12:**
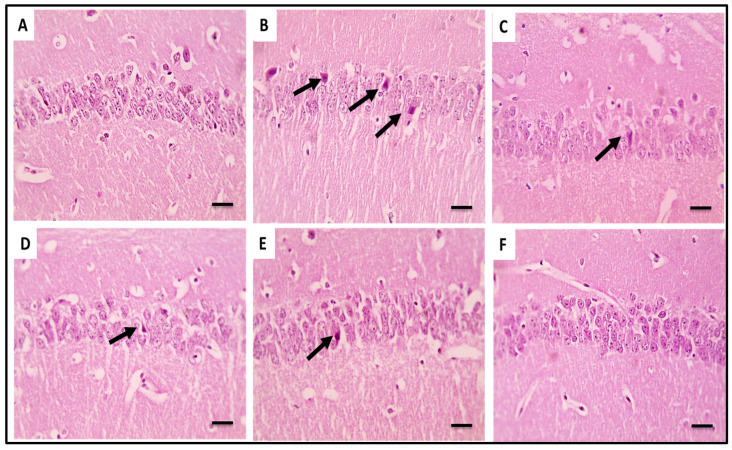
H&E-stained brain sections showing the hippocampus of the different groups. Normal neurons were revealed in the control group (group 1, (**A**)). Sections from the group that received LPS (group 3, (**B**)) showed the appearance of many degenerated neurons in the pyramidal layer (black arrows). Sections from the treated group with RVS (group 4, (**C**)), SPE 100 mg/kg (group 5, (**D**)), and SPE 200 mg/kg (group 6, (**E**)) showed the presence of a few degenerated neurons in the pyramidal layer (black arrows). Sections from the treated group with SPE 300 mg/kg showed normal neurons in the pyramidal layer (group 7, (**F**)) (×400).

Figure 10, Figure 11 and Figure 12 depict brain sections stained with H&E that illustrate the histopathological characteristics of the various groups in the cerebral cortices, cerebellum, and hippocampus. In the H&E-stained cerebral cortices sections of group 5, there were normal neurons with congested blood vessels. Group 6 exhibited normal neurons with mildly congested blood vessels, while the group 7 sections revealed completely normal neurons. In addition, in the H&E-stained cerebellum sections of group 5, there were markedly degenerated purkinje neurons. Group 6 exhibited a few degenerated purkinje neurons, while the group 7 sections revealed completely normal purkinje neurons. In addition, in the H&E-stained hippocampus sections of groups 5 and 6, there were a few degenerated neurons in the pyramidal layer. Group 7 revealed completely normal neurons in the pyramidal layer.

## 3. Discussion

Neurological disorders are a significant challenge for healthcare systems all over the world. Due to the stressful nature of the current era, these diseases are unfortunately increasing and affecting a large number of people. In addition, treatment options for these diseases are scarce or nonexistent. Most of the used drugs merely alleviate the disease symptoms [22]. Consequently, the discovery of new therapeutic agents for these neurologic diseases is crucial. Plants are considered an abundant source of varieties of bioactive phytochemicals [23], which can be utilized in the treatment of neurological disorders.

In the negative and positive ESI modes, the LC-ESI-MS/MS analysis of SPE revealed 36 compounds of phenolic glycosides, coumaric acids, flavonoids, alkyl glucosinolate, and indoles. Diosmin, isosakuranetin-7-*O*-neohesperidoside (poncirin), acacetin-7-*O*-rutinoside, rhoifolin, agmatine, benzyl glucosinolate, pantothenate, spermidine, hydroxybenzoic acid, malic acid, and 3-formylindole are the most abundant compounds. The neuroprotective effect of diosmin [24] and rhoifolin [25] has been reported in the literature, which may explain the neuroprotective effect of SPE in the current study. In this study, it was found that the SPE contained benzyl glucosinolate. It was revealed that glucosinolate-derived isothiocyanate as benzyl glucosinolate exhibited a broad range of activities against the onset and progression of several neurodegenerative disorders. Glucosinolates have no significant biological effect, but isothiocyanates produced from the hydrolysis of glucosinolates have a neuroprotective effect. The hydrolysis of glucosinolates may be catalyzed by thioglucosidase enzymes present in the gut microbiota of mice, as in humans [26,27]. The ability of isothiocyanates, the activated form of glucosinolate compounds, to improve the endogenous degradation of the protein systems has been investigated as a key protective mechanism to oppose the anomalous build-up of toxic protein oligomers in most neurodegenerative diseases [28]. The reported neuroprotective effect of indole-3-carbinol in PA disease [29] could be used to infer the potential neuroprotective effect of 3-formylindole [29].

Flavonoid-rich foods are significant nutraceuticals that improve health and cognitive capabilities, delay aging, combat chronic diseases, increase life expectancy, and prevent or slow the pathological symptoms of neurodegenerative disorders. Flavonoids may suppress cholinesterases, including AChE, with free-radical scavenging effects and modulation of the signaling pathways included in the cognitive and neuroprotective functions. They increase the vascular blood flow and stimulate neurogenesis. Flavonoids also inhibit apoptosis in neuronal cells [30].

Pantothenate counteracted the pathological effects by preventing disturbances in the glutathione system [31]. Spermidine is a polyamine and an autophagy inducer that can maintain neuronal homeostasis. Healthy brain development and function are dependent on the brain’s polyamine concentration. Polyamines interact with the opioid system, glutamatergic signaling, and neuroinflammation in the neuronal and glial compartments. Among the polyamines, spermidine is found at the highest level in the human brain. Age-linked fluctuations in spermidine levels may contribute to impairments in neural networks and neurogenesis. Spermidine has antiaging and anti-inflammatory properties that give protection against neurotoxicity and neurological disorders. Therefore, a polyamine-rich plant extract may be a promising target for increasing the spermidine levels in the brain [32].

HSV-2 is one of the viruses that cause neurological disorders. Therefore, we determined the in vitro antiviral activity of SPE against HSV-2 by using the plaque assay. This assay is a standard quantitative method for determining the number of infectious viruses [33]. After allowing serial dilutions of the tested virus to infect cells in the cell culture, this is accomplished by counting the plaques that form in the cell culture [34]. In this study, SPE demonstrated antiviral activity against HSV-2 with a IC_50_ of 8.946 ± 0.02 µg/mL. The value of IC_50_ represents the concentration of the tested compound needed to produce 50% inhibition of the studied viruses [34].

Neuroinflammation has been hypothesized to play a role in the development of cognitive impairment and neurodegenerative disorders. Several neurodegenerative diseases, including AD, PA, amyotrophic lateral sclerosis (ALS), and MS (multiple sclerosis), and their specific pathophysiology are unknown. For the study of cognitive impairment associated with neuroinflammation and neurodegenerative diseases, the development of a suitable animal model is essential.

This study aimed to determine how SPE protects mice with LPS-induced neuroinflammation from memory loss and immune reactions. Several studies have demonstrated that the administration of LPS causes neuroinflammation and blood–brain barrier (BBB) damage, followed by amyloid formation and memory loss. In addition, neuronal loss and microglial activation brought on by LPS-induced brain inflammation caused the release of neurotoxic substances, including inflammatory cytokines (TNF-α and IL-6). Chronic LPS administration can impair learning and spatial memory, such as the cognitive loss seen in AD, which is linked to inflammation and amyloid formation due to increased β-amyloid protein (Aβ) deposition [35,36,37].

The mechanism by which SPE improved LPS-induced neuroinflammation may be explained by its direct action on the brain via antioxidant and anti-inflammatory effects. The cholinergic system in neuroinflammatory disorders is unbalanced [38]. AChE inhibitors enhance cognition [39] and are the most effective therapy for treating AD patients [40]. LPS increased the activity of AChE in mice brains, which is consistent with a previous study by Tyagi et al. [41]. It caused oxidative damage, decreased acetylcholine (Ach) in the brain [42], and disrupted the cholinergic system by ROS induction [43]. Age-related dementia is correlated with the increase in oxidative stress in the aged population [44]. According to the results of the current experiment, pretreatment with SPE improved the mice’s cognitive performance as the SPE (300 mg/kg)-treated group showed a substantial decrease in AChE activity compared to the LPS-treated group.

In addition, H&E-stained brain sections alleviated the detrimental effects of LPS in the cerebral cortices, cerebellum, and hippocampus in the group pretreated with 300 mg/kg SPE.

The process of inflammation is caused by the activation of many inflammatory pathways [45], and several inflammatory mediators, such as iNOS, IL-6, TNF-α, and proapoptotic markers (caspase-3 and c-Jun) are released in this process [46]. Due to its high aerobic metabolism, blood perfusion, and deficient antioxidant defense, the brain is highly susceptible to oxidative damage. As a result, cognitive decline and neuronal damage are attributed to oxidative stress in the brain [47]. Previous research has shown that a mouse’s brain underwent a considerable change in oxidative stress indicators after receiving an injection of LPS [48].

Our study found that SPE reduced the proinflammatory mediators and proapoptotic markers in the LPS-induced mice. The MDA levels significantly increased (*p* ≤ 0.05) in the brains of the LPS group compared to the normal control group, whereas the CAT and SOD levels significantly decreased (*p* ≤ 0.05). Additionally, our findings revealed that SPE administration reversed the aberrant changes in the MDA, SOD, and CAT levels induced by LPS administration. This result demonstrates that the neuroprotective properties of SPE can be attributed to its antioxidant potential. These findings are supported by earlier studies that revealed the ROS-scavenging effects of *S. persica* [48,49].

TNF-α stimulates the production of other cytokines, including IL-6, IL-1, IL-3, etc. Increased Aß deposition is linked to the activation of these cytokines. According to several studies, IL-6 and TNF-α are raised by LPS treatments [50,51]. The current research shows that LPS-treated mice have higher TNF-α, IL-6, iNOS, caspase-3, and c-Jun markedly decreased by SPE and rivastigmine pretreatment. Numerous in vivo investigations have suggested that inhibiting TNF-α and IL-6 may enhance long-term potentiation and postpone or prevent neuronal dysfunction [52,53]. It has been found that an increase in the caspase-3 levels is related to TNF downstream signaling. These results are supported by research demonstrating a role for caspase-3 in neurodegeneration. Evidence suggests that caspase-3 degrades the tau protein and produces neurofibrillary tangles that impair cognitive function [54]. Similar findings were found in our investigation. SPE administration decreased the LPS-induced production of proinflammatory cytokines, such as TNF-α, IL-6, and iNOS, and apoptotic markers, such as caspase-3 and c-Jun. Hence, SPE prevented LPS-induced neuroinflammation, which prevented memory loss and enhanced cognition. The phosphorylation of tau proteins that causes plaque formation is another process involving JNK 3. As previously reported, c-Jun is the JNK pathway’s downstream effector [55], and LPS can activate the c-Jun/JNK pathway, which promotes apoptosis [56]. Our results are consistent with these findings, as the LPS-treated group had elevated c-Jun levels, markedly decreasing the administration of SPE and RVS. As a result, it was proven that SPE had an impact on the apoptosis-related protein c-Jun.

## 4. Materials and Methods

### 4.1. Chemicals and Media

All chemicals and media were bought from Merck, the UK, and Oxoid, USA.

### 4.2. Plant Collection and Preparation of Extract

The fresh stems and twigs barks of *S. persica* shrubs were collected in June 2021 from a farm of Tahoor Freshler Meswak at El-Bagor, Monufia Governorate, Egypt. The plant was identified by Prof. Dr. Mohammed Ibrahim Fotoh, Professor of Ornamental Horticulture and Landscape Design, at the Faculty of Agriculture, Tanta University. The fresh stems and twigs barks (1.5 kg) were dried at room temperature, ground, and extracted with 95% methanol (4 L × 3 times). The combined extract was evaporated at 40 °C under vacuum to obtain 46.7 g of the extract as a dry residue. A representative sample was kept (PG-A-00522) at the Herbarium of the Department of Pharmacognosy, Faculty of Pharmacy, Tanta University.

### 4.3. LC-ESI-MS/MS Analysis of SPE

Using LC-ESI-MS/MS and the methodology previously described, the metabolic profile of the defatted methanol extract of *S. persica* stem bark was determined [57,58].

### 4.4. In Vitro Antiviral Potential

#### 4.4.1. Viruses and Cell Lines

Vero-E6 cells (Vacsera, Cairo, Egypt) were utilized in the propagation of herpes simplex virus type 2 (HSV-2) in Dulbecco’s modified Eagle’s medium (DMEM) with 10% fetal bovine serum (FBS) and 1% penicillin/streptomycin antibiotics. The viral stocks were generated by inoculating the cells with the viruses in tissue culture flasks and incubating them in an atmosphere containing 5% CO_2_ at 37 °C. The tissue culture flasks were inoculated with Vero-E6 cells a day before the virus infection. Trypsin (1%) was added to the infection medium after treating L-1-tosylamido-2-phenylethyl chloromethyl ketone. After two hours of incubation, the medium with the viruses was removed, and a new medium was added and incubated for three days. Following centrifugation to remove cell debris, the supernatant was aliquoted and titrated using the plaque assay [59].

#### 4.4.2. MTT Cytotoxicity Assay

Using 3-(4,5-dimethylthiazol-2-yl)-2,5-diphenyltetrazolium bromide (MTT), the half-maximal cytotoxic concentration (CC50) of SPE was determined [59,60]. The absorbance (A) of the produced formazan was identified at an optical density (OD) of 540 nm using an ELISA reader (Sunrise Tecan, Zürich, Switzerland). The cytotoxicity percentage was calculated according to the equation:

$$\%\;\mathrm{cytotoxicity}=\frac{\;A\;\left(\mathrm{cells}\;\mathrm{without}\;\mathrm{treatment}\right)-A\;\left(\mathrm{treated}\;\mathrm{cells}\right)}{A\;\left(\mathrm{cells}\;\mathrm{without}\;\mathrm{treatment}\right)\;}\times 100$$

#### 4.4.3. Plaque Assay

As previously reported [24], it was performed to elucidate the potential antiviral SPE against HSV-2 in a six-well tissue culture plate seeded with Vero-E6 cells (90% confluent). Briefly, after the propagation of the virus, as mentioned earlier, it was 10-fold serially diluted using DMEM. After that, the diluted virus suspension (100 µL) was mixed with DMEM (400 µL), added to the tissue culture plate wells, and incubated at 37 °C. After one hour, the suspension was removed, and the Vero-E6 cells were covered with DMEM supplied with 2% agarose (2%) and the SPE at nontoxic concentrations. The agarose was allowed to solidify, and the tissue culture plates were incubated at 37 °C for 72 h. Subsequently, 10% formalin was added to the well for one hour, removed, and the cells were washed. Finally, 0.1% crystal violet solution was added for staining, and then, the cells were rinsed with water and left to dry. The plaques were enumerated as nonstained spots with a violet background, and the percentage of the inhibition of the plaque formation was calculated using the following equation:

$$\%\;\mathrm{inhibition}=\frac{\mathrm{Untreated}\;\mathrm{viral}\;\mathrm{count}-\mathrm{treated}\;\mathrm{viral}\;\mathrm{count}}{\mathrm{untreated}\;\mathrm{viral}\;\mathrm{count}}\times 100$$

### 4.5. In Vivo Protective Effect against LPS-Induced Neuroinflammation in Mice

#### 4.5.1. Animals

Forty-two adult albino male mice (22–34 g) were used in this study. The animals were maintained in standard laboratory conditions, including 25 ± 2 °C, 60–70% humidity, and free access to food and water. The animals were acclimated for ten days before the trial started [61].

#### 4.5.2. Experimental Groups

The animals were divided randomly into seven groups of six mice each.

Group 1 (vehicle control): mice were administered 0.2 mL saline intraperitoneally (IP).

Group 2: administered only 300 mg/kg of SPE without LPS administration.

Group 3 (negative control or LPS group): mice were administered 0.03 mL of LPS (0.25 mg/kg, IP).

Group 4 (Rivastigmine or RVS group): mice administered RVS (0.25 mg/kg, IP).

Groups 5, 6, and 7: administered SPE in saline at 100, 200, and 300 mg/kg orally.

The treatment period was 28 days, and LPS was administered from days 15 to 21 (groups 3–7). The animals were euthanized on the last day of the previously mentioned period.

#### 4.5.3. Biochemical Assessment

The brains were immediately removed and placed in an extremely cold isotonic solution. The brain was homogenized in phosphate-buffered saline (PBS, pH 7.4). The homogenate was centrifuged at 10,000× *g* for 15 min, and the supernatant was used for further biochemical analysis.

##### 4.5.3.1. Evaluation of Acetylcholinesterase (AChE) Activity

The activity of AChE reflects both the imbalance in the cholinergic system, as well as the degeneration of the cholinergic neurons in the brain. Briefly, 3 mL of sodium phosphate buffer (0.01 M, pH 8), 50 mL of the supernatant from the brain tissue homogenate, 100 mL of acetyl thio-choline iodide (AcSCh, 0.75 nM), and 100 mL of 5,5′-dithiobis-(2-nitrobenzoic Acid) (DTNB or Ellman’s reagent, 10 mM) were mixed. At 412 nm, the absorbance was measured spectrophotometrically every 30 s for 5 min. Micromoles of AcSCh hydrolyzed per min per mg of protein were used to express the results [62].

##### 4.5.3.2. Estimation of Malondialdehyde (MDA) Level

The thiobarbituric acid reactive substances (TBARS) assay was used to quantify MDA, as it can detect the products of lipid oxidation (LPO), as TBARS acts as a source of ROS. This procedure involved incubating 0.1 mL of the brain supernatant with 0.5 mL of Tris-HCl (0.1 M, pH 7.4) for two hours. Following incubation to precipitate the proteins, one milliliter of trichloroacetic acid (TCA) solution (10% *w*/*v*) was added. The combined product was centrifuged for 10 min with 1000× *g* at 4 °C. The supernatant was decanted, 0.67% thiobarbituric acid (TBA) was added, and the mixture was placed in boiling water for 15 min. The tubes were immediately placed in an ice bath to cool for 15 min, and then, 1 mL of distilled water was added. A spectrophotometer determined the amount of MDA at 532 nm [63].

##### 4.5.3.3. Estimation of Superoxide Dismutase (SOD) Level

The ability of the enzyme to remove the superoxide radicals created by pyrogallol in an alkaline solution was the basis for measuring the SOD activity. Briefly, 180 mL of potassium phosphate buffer (0.1 M, pH 7.4) and 10 mL of pyrogallol solution were added to 10 L of brain tissue homogenate (2.6 mM in 10 mM HCl). For five minutes, the absorbance increase rate measurements were taken every 30 s. The enzyme needed to block pyrogallol autoxidation by 50% per 200 L of the assay mixture was used to define one unit of SOD [64].

##### 4.5.3.4. Estimation of Catalase (CAT) Level

The CAT level was determined was performed using a biodiagnostics kit (CA 2517, Biodiagnostics, Cairo, Egypt). In brief, the catalase enzyme was allowed to react with a known quantity of H_2_O_2_, and after one minute, the reaction was stopped using a catalase inhibitor. The remaining H_2_O_2_ was combined with 3,5-dichloro-2-hydroxybenzene sulfonic acid (DHBS) and 4-aminophenazone (AAP) in the presence of horseradish peroxidase (HRP) to create a chromophore with a color intensity that was inversely proportional to the quantity of catalase in the initial sample [65].

##### 4.5.3.5. Determination of Caspase-3, Interleukin-6 (IL-6), Tumor Necrosis Factor-Alpha (TNF-α), Inducible Nitric Oxide Synthase (iNOS), and c-Jun Genes Expression by Quantitative Real-Time PCR (qRT-PCR)

TRIzol reagent (Invitrogen, Waltham, USA) was used to purify RNA from the tissue samples. The Maxima first strand cDNA synthesis kit (Thermo Scientific, Waltham, MA, USA) was then used to create complementary DNA (cDNA). The primer sets for genes were produced using the Primer3PLUS program (version 0.4.0; available at http://frodo.wi.mit.edu (accessed on 1 December 2022); Table S1). According to the procedure outlined [66], real-time PCR experiments were carried out using the Applied Biosystem 7500 real-time PCR detection system (Life Technologies, San Francisco, CA, USA) using SensiFAST Sybr green Low-Rox PCR master mix kit (Bioline, London, UK). The housekeeping gene was β-actin. In the control group, the relative gene expression for each gene was adjusted to one (negative control) [67].

### 4.6. Histopathological Analysis

The collected tissue specimens from each group were immediately preserved in 10% formalin. Hematoxylin and eosin (H&E) stains were used to examine the brain sections histopathologically under a light microscope [68].

### 4.7. Statistics

The results obtained were expressed as mean ± standard deviation (SD). The in vitro antiviral study used ANOVA and post hoc test (Tukey). Statistical analysis of the in vivo study of each variable was tested for normality using the Shapiro–Wilk normality test. The differences in each variable were analyzed using Kruskal-Wallis, followed by Dunn’s multiple comparisons tests. Statistical analysis was conducted using GraphPad Prism (version 9.3.1, GraphPad, San Diego, CA, USA). The level of statistical significance was set at (*p* < 0.05).

## 5. Conclusions

The LC-ESI-MS/MS analysis of SPE tentatively demonstrated the diversity of its bioactive compounds. Diosmin, isosakuranetin-7-*O*-neohesperidoside, acacetin-7-*O*-rutinoside, rhoifolin, spermidine, pantothenate, benzyl glucosinolate, hydroxybenzoic acid, and 3-formylindole were the most abundant compounds. These components may account for *Salvadora persica*’s anti-inflammatory and antioxidant properties. This study demonstrated that SPE possessed antiviral activity against HSV-2, the causative agent of certain neurological disorders. Additionally, SPE reduced the brain inflammation caused by LPs. The decrease in the MDA levels and the increase in the SOD and CAT levels demonstrated the ability of SPE to inhibit oxidative stress. It exhibited anti-neuroinflammatory and antiapoptotic properties by decreasing the LPS-induced production of proinflammatory cytokines such as TNF-, IL-6, and iNOS. SPE enhanced the cognitive performance of mice due to a significant decrease in AChE activity compared to the LPS-treated group. At 300 mg/kg of SPE, normal neurons appeared in the cerebral cortices, cerebellum, and hippocampus. Therefore, it should be considered that SPE could be used for prophylaxis and to treat neurodegenerative diseases caused by inflammatory or oxidative damage.

## Figures and Tables

**Table 1:** LC-ESI-MS/MS analysis of the methanol extract of *Salvadora persica* stem bark displaying the tentatively recognized compounds.

#	RT (min)	Precursor m/z	Error ppm	Compound Name	Formula	Adduction Name	MS/MS	Ontology
1	1.1	131.129	0	Agmatine	C_5_H_14_N_4_	[M + H]^+^	60.0550, 72.0801, 114.0965	Guanidines
2	1.12	146.165	0.3	Spermidine	C_7_H_19_N_3_	[M + H]^+^	72.0794, 84.08425, 112.1084, 129.1375	Dialkylamines
3	1.15	133.014	−0.6	Malic acid	C_4_H_6_O_5_	[M − H]^−^	71.0144, 72.9925, 89.0248, 115.0042	Beta hydroxy acids derivatives
4	1.17	173.045	−0.8	Shikimic acid	C_7_H_10_O_5_	[M − H]^−^	73.0265, 93.0345, 111.0066, 138.9140, 154.9062	Shikimic acids derivatves
5	1.23	104.107	1.2	Choline	C_5_H_14_NO	[M]^+^	58.0660, 60.0816	Cholines
6	1.64	124.039	0.3	Nicotinic acid	C_6_H_5_NO_2_	[M + H]^+^	78.0352, 80.05060, 107.0404	Pyridine carboxylic acids
7	1.65	86.096	−0.3	Piperidine	C_5_H_11_N	[M + H]^+^	69.0710, 86.0969	Piperidines
8	2.06	408.042	0.7	Benzyl glucosinolate	C_14_H_19_NO_9_S_2_	[M − H]^−^	74.9906, 95.9517, 166.0331, 241.0030, 259.0139, 274.9913	Alkylglucosinolates
9	2.33	220.118	−0.6	Pantothenate	C_9_H_17_NO_5_	[M + H]^+^	90.0526, 202.1076	Secondary alcohols
10	2.75	167.035	−0.7	Homogenentisic acid	C_8_H_8_O_4_	[M − H]^−^	108.0210, 109.0304, 122.0345, 123.0485	2(hydroxyphenyl) acetic acids
11	2.93	137.024	−1.8	*P*-hydroxybenzoic acid	C_7_H_6_O_3_	[M − H]^−^	65.0400, 75.0232, 93.0337	Hydroxybenzoic acid derivatives
12	4.38	390.175	−0.3	Syringin	C_17_H_24_O_9_	[M + NH_4_]^+^	105.0682, 133.0636, 161.0610, 193.0851, 211.0949	Phenolic glycosides
13	4.69	359.077	−0.3	Rosmarinic acid	C_18_H_16_O_8_	[M − H]^−^	72.9911, 123.0408, 133.0296, 161.0247, 179.0321, 197.0480	Coumaric acids derivatives
14	6.26	223.061	0.1	Sinapic acid	C_11_H_12_O_5_	[M − H]^−^	93.0322, 121.0278, 149.0237, 177.0510, 193.0141, 205.0518	Hydroxycinnamic acids
15	6.51	593.151	0.7	Kaempferol-7-*O*-neohesperidoside	C_27_H_30_O_15_	[M − H]^−^	284.0328, 385.0439	Flavonoid-7-*O*- glycosides
16	6.54	596.173	−0.1	cyanidin-3-*O*- rutinoside	C_27_H_31_O_15_	[M]^+^	287.0549, 449.1057	Anthocyanidin-3-*O*-glycosides
17	6.74	301.071	−0.4	Hesperetin	C_16_H_14_O_6_	[M − H]^−^	269.0488, 289.0489, 301.0726	4′-*O*-methylated flavonoids
18	6.81	181.050	0.7	Syringaldehyde	C_9_H_10_O_4_	[M − H]^−^	67.0180, 123.0072, 151.0023, 166.0266	Methoxyphenols
19	6.87	167.035	−0.4	5-Methoxysalicylic acid	C_8_H_8_O_4_	[M − H]^−^	108.0208, 124.0111, 152.0111	M-methoxybenzoic acids derivatives
20	7.23	579.170	−0.2	Rhoifolin	C_27_H_30_O_14_	[M + H]^+^	271.0579, 433.1155	Flavonoid-7-*O*- glycosides
21	7.54	609.181	−0.6	Diosmin	C_28_H_32_O_15_	[M + H]^+^	286.0526, 301.0743, 463.1243	Flavonoid-7-*O*- glycosides
22	7.63	494.141	−0.2	Malvidin-3- galactoside	C_23_H_25_O_12_	[M]^+^	137.0616, 163.0757, 253.0856, 285.1151, 313.1100, 331.1161	Anthocyanidin-3-*O*-glycosides
23	7.94	174.056	0	1-methoxyindole-3-carbaldehyde	C_10_H_9_NO_2_	[M − H]^−^	131.0379, 159.0321	Indoles
24	8.02	137.132	−5	Sabinene	C_10_H_16_	[M + H]^+^	65.0379, 94.0376, 122.0327	Bicyclic monoterpenoids
25	8.12	144.045	−1	3-Formylindole	C_9_H_7_NO	[M − H]^−^	114.0339, 115.0422, 116.0502, 126.0343, 142.0292	Indoles
26	8.29	177.055	−0.9	Coniferaldehyde	C_10_H_10_O_3_	[M − H]^−^	129.0025, 134.0377, 162.0320	Methoxyphenols
27	8.61	163.040	−0.8	2-Hydroxy cinnamic Acid (2-Coumaric acid)	C_9_H_8_O_3_	[M − H]^−^	76.09749, 92.0297, 93.0360, 120.0197	Hydroxycinnamic acids
30	9.08	593.186	−0.2	Acacetin-7-*O*- rutinoside	C_28_H_32_O_14_	[M + H]^+^	85.0299, 129.0558, 242.0574, 285.0782, 447.1322	Flavonoid-7-*O*- glycosides
31	9.12	591.171	0.2	Acacetin-7-*O*- neohesperidoside	C_28_H_32_O_14_	[M − H]^−^	268.0378, 283.0612	Flavonoid-7-*O*- glycosides
32	9.27	595.202	0.8	Isosakuranetin-7-*O*-neohesperidoside	C_28_H_34_O_14_	[M + H]^+^	85.0306, 129.0552, 153.0207, 195.0301, 263.0574, 287.0930, 433.1550, 449.1382	Flavonoid-7-*O*- glycosides
33	9.28	287.091	0.7	Isosakuranetin	C_16_H_14_O_5_	[M + H]^+^	91.0548, 153.0187, 161.0641	4′-*O*-methylated flavonoids
34	9.78	447.128	0.1	Sissotrin	C_22_H_22_O_10_	[M + H]^+^	149.0152, 242.0657, 270.0481, 285.0748	Isoflavonoid *O*-glycosides
35	10.51	269.045	0.5	Apigenin	C_15_H_10_O_5_	[M − H]^−^	117.0353, 148.0222, 151.0004, 254.0616	Flavones
36	14.00	285.075	−0.2	Acacetin	C_16_H_12_O_5_	[M + H]^+^	153.0229, 187.0495, 242.0585, 270.0551	4′-*O*-methylated flavonoids

## Data Availability

The authors confirm that the data supporting this study are available within the article and Supplementary Files.

## Supplementary Materials

The following supporting information can be downloaded at: https://www.mdpi.com/article/10.3390/ph16030398/s1: Figure S1: Total ion chromatogram (negative mode) of LC-ESI-MS/MS of methanol extract of *Salvadora persica* stem bark. Figure S2: Total ion chromatogram (positive mode) of LC-ESI-MS/MS of methanol extract of *Salvadora persica* stem bark. Table S1: Primer sequences of the tested genes.
