# Supplementary material for: In Vitro Antiviral Effect and Potential Neuroprotection of Salvadora persica L. Stem Bark Extract against Lipopolysaccharides-Induced Neuroinflammation in Mice: LC-ESI-MS/MS Analysis of the Methanol Extract
Source: Pharmaceuticals (Basel). 2023 Mar 6;16(3):398. doi: 10.3390/ph16030398 (PMC10058283; doi:10.3390/ph16030398)

In Vitro Antiviral Effect and Potential Neuroprotection of *Salvadora persica* L. Stem Bark Extract against Lipopolysaccharides-Induced Neuroinflammation in Mice: LC-ESI-MS/MS Analysis of the Methanol Extract

Reem Binsuwaidan [1,†], Walaa A. Negm [2,*,†], Engy Elekhnawy [3,*], Nashwah G. M. Attallah [4], Eman Ahmed [5,6], Sameh Magdeldin [6,7], Ehssan Moglad [8], Sally Abdallah Mostafa [9] and Suzy A. El-Sherbeni [2]

1 Department of Pharmaceutical Science, College of Pharmacy, Princess Nourah Bint Abdulrahman University, P.O. Box 84428, Riyadh 11671, Saudi Arabia
2 Department of Pharmacognosy, Faculty of Pharmacy, Tanta University, Tanta 31527, Egypt
3 Department of Pharmaceutical Microbiology, Faculty of Pharmacy, Tanta University, Tanta 31527, Egypt
4 The Egyptian Drug Authority (EDA), Previously NODCAR, Giza 8655, Egypt
5 Department of Pharmacology, Faculty of Veterinary Medicine, Suez Canal University, Ismailia 41522, Egypt
6 Proteomics and Metabolomics Research Program, Department of Basic Research, Children's Cancer Hospital 57357, Cairo 11441, Egypt
7 Department of Physiology, Faculty of Veterinary Medicine, Suez Canal University, Ismailia 41522, Egypt
8 Department of Pharmaceutics, College of Pharmacy, Prince Sattam Bin Abdulaziz University, P.O. Box 173, Al-Kharj 11942, Saudi Arabia
9 Department of Medical Biochemistry and Molecular Biology, Faculty of Medicine, Mansoura University, Mansoura 35516, Egypt
* Correspondence: walaa.negm@pharm.tanta.edu.eg (W.A.N.); engy.ali@pharm.tanta.edu.eg (E.E.)
† These authors contributed equally to this work.

Table S1. Primer sequences of the tested genes.

Gene	Primer Sequences	Reference Sequences	PCR product size (bp)
**β-actin**	F: CTGGCTCCTAGCACCATGAA<br>R: AGCTCAGTAACAGTCCGCCTA	NM_007393.5	189bp
**Caspase 3**	F: AGCTTGGAACGGTACGCTAAG<br>R: AGTCCACTGACTTGCTCCCA	NM_001284409.1	116bp
**IL- 6**	F: ACCCCAATTTCCAATGCTCTCC<br>R: ATAACGCACTAGGTTTGCCGA	NM_031168.2	146bp
**TNF-α**	F: CCACCACGCTCTTCTGTCTA<br>R: CCACTTGGTGGTTTGTGAGTG	NM_001278601.1	126bp
**iNOS**	F: GGTGAAGGGACTGAGCTGTTA<br>R: TGAAGAGAAACTTCCAGGGGC	NM_010927.4	163 bp
**c-Jun**	F: CCGAGAATTCCGTGACGACT<br>R: TAGCACTCACGTTGGTAGGC	NM_010591.2	160BP

Figure S1: Total ion chromatogram (negative mode) of LC-ESI-MS/MS of methanol extract of *Salvadora persica* stem bark

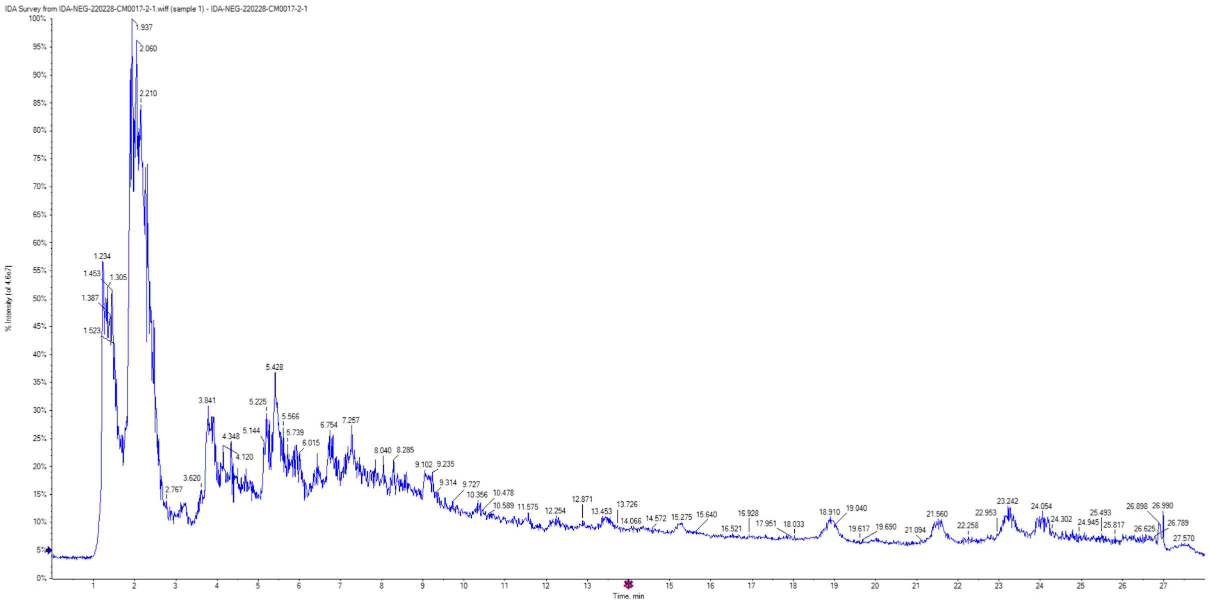

Figure S2: Total ion chromatogram (positive mode) of LC-ESI-MS/MS of methanol extract of *Salvadora persica* stem bark

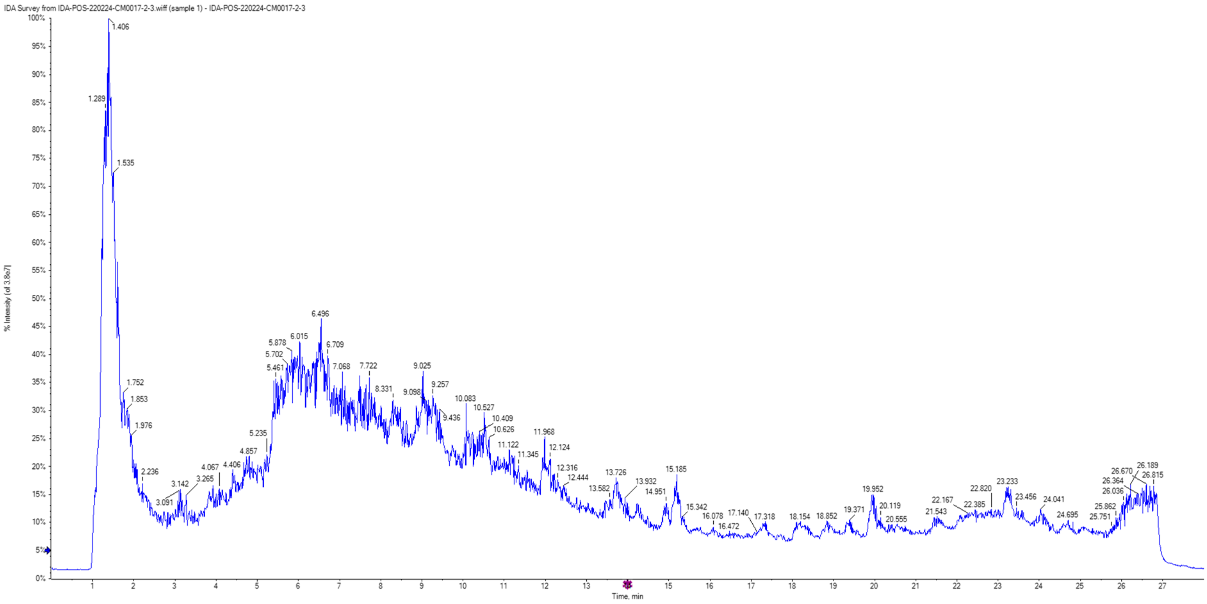

Supplement: Supplementary file 1 [file pharmaceuticals-16-00398-s001.zip › pharmaceuticals-2201807-supplementary.pdf]
